# Insights into the meaning of medical students’ studies. An online survey at two medical faculties

**DOI:** 10.3205/zma001700

**Published:** 2024-09-16

**Authors:** Felix Albrecht, Gabriele Lutz, Gina Atzeni, Pascal O. Berberat, Paula Matcau, Nana Jedlicska, Claudia Kiessling

**Affiliations:** 1 Witten/Herdecke University, Faculty of Health, Witten, Germany; 2 Witten/Herdecke University, Faculty of Health, Psychosomatic Medicine and Psychotherapy, Witten, Germany; 3 Gemeinschaftskrankenhaus Herdecke, Herdecke, Germany; 4 LMU Munich, Institute of Sociology, Munich, Germany; 5 Technical University of Munich, TUM School of Medicine and Health, Department of Clinical Medicine, TUM Medical Education Center, Munich, Germany; 6 Witten/Herdecke University, Faculty of Health, Education of Personal and Interpersonal Competencies in Health Care, Witten, Germany

**Keywords:** meaning in life, search for meaning, meaning crises, medical education

## Abstract

**Objective:**

The aim of the study was to investigate how medical students’ deal with their own questions of meaning during their studies, how they cope with patients’ questions of meaning or crises of meaning, to what extent their experience of meaning changes during their studies, and what role medical studies play in this.

**Methods:**

In 2022, we conducted an exploratory cross-sectional study in the form of an online survey at two German universities with students in the clinical part of their studies. Quantitative data were analyzed descriptively, and group differences were analyzed using Mann-Whitney U tests. Free-text comments were analyzed using thematic analysis.

**Results:**

Of the 111 participants (response rate 12%), 92% had addressed questions of meaning. 64% of the students felt that their studies were meaningful, and 45% felt that their clinical internships were meaningful. 59% reported that they had been confronted with questions of meaning in their contact with patients, although many of them felt that they had been inadequately prepared for this (56%). This impression was stronger among respondents at the beginning of the clinical phase compared to respondents at the end (U(56,34)=660, p=0.012). According to the students, strategies for dealing with questions of meaning were active engagement with topics of meaning, tolerance of uncertainties, or avoidance. In addition to the basic requirement of openness to all topics of meaning, students expressed the wish to be better prepared for professional questions of meaning and for follow-up work on stressful events. A wide range of critical experiences with training and the healthcare system had an inhibiting effect on the experience of meaning.

**Conclusion:**

Since a higher sense of purpose can be associated with improved health and motivation, university programs might have the potential to support students’ sense of purpose and, in the long term, improve their capacities to support patients who grapple with questions of meaning.

## Zusammenfassung

**Zielsetzung:**

Ziel der Studie war es zu untersuchen, wie Medizinstudierende mit eigenen Sinnfragen im Studium umgehen, wie sie Sinnfragen oder -krisen von Patient*innen bewältigen, inwieweit sich Sinnerleben im Laufe des Studiums ändert und welche Rolle das Medizinstudium dabei spielt.

**Methoden:**

Wir führten 2022 eine explorative Querschnittstudie in Form einer Onlinebefragung an zwei deutschen Universitäten mit Studierenden im klinischen Studienabschnitt durch. Quantitative Daten wurden deskriptiv, Gruppenunterschiede mit Mann-Whitney U Tests analysiert. Freitextkommentare wurden mittels thematischer Analyse ausgewertet.

**Ergebnisse:**

Von den 111 Teilnehmenden (Rücklaufquote 12%) hatten sich 92% mit Sinnfragen auseinandergesetzt. Das Studium wurde von 64% als sinnstiftend empfunden, die klinischen Praktika von 45%. Im Patient*innenkontakt gaben 59% an, mit Sinnfragen konfrontiert worden zu sein, wobei sich viele nur unzureichend (56%) darauf vorbereitet fühlten. Dieses Gefühl war bei den Befragten zu Beginn der klinischen Phase höher als bei den Befragten am Ende (*U*(56,34)=660, *p*=0,012). Strategien im Umgang mit Sinnfragen waren laut den Studierenden die aktive Auseinandersetzung mit Sinnthemen, das Aushalten von Ungewissheiten oder Vermeidung. Die Studierenden äußerten neben der Grundvoraussetzung einer Offenheit aller gegenüber Sinnthemen, den Wunsch nach einer besseren Vorbereitung auf berufliche Sinnfragen und Nachbereitung von belastenden Ereignissen. Vielfältige kritisch empfundene Erfahrungen mit der Ausbildung und dem Gesundheitssystem hemmten das Sinnerleben.

**Schlussfolgerung:**

Da höheres Sinnerleben mit verbesserter Gesundheit und Motivation einhergehen kann, könnten universitäre Angebote das Potenzial haben, das Sinnerleben von Studierenden zu unterstützen und langfristig die Begleitung von Patient*innen mit Sinnfragen zu fördern.

## 1. Introduction

The search for meaning in life is a significant topic for many people [1]. Terms such as meaning, meaning search, or meaning experience are often used in everyday language. Academic literature defines meaning experience in many different ways. Despite different approaches, the definitions according to Martela and colleagues have three general similarities: *meaning, purpose and coherence* [2]. On the one hand, the experience of meaning can be composed of the subjective *meaning* that a person attaches to a thing, action or event in a particular situation [3]. On the other hand, it can be composed of a higher *purpose*, so that the meaning of life is geared towards achieving goals [4], and of a sense of *coherent connectedness* with oneself and the environment, also known as coherence [5], [6], [7], [8]. While the experience of meaning seems to be an everyday, concrete [9], [10] and unconscious evaluation process, a conscious examination of meaning topics is usually preceded by an external trigger [4]. Sources of meaning are individual and diverse. For example, many people find social interaction, group experiences, spiritual moments, or nature experiences meaningful [4], [11]. 

It is only in the last 25 years that researchers have focused on the meaning of life in empirical studies in the fields of psychology, medicine, and education [4], [11], [12], [13]. Various studies have shown that a strong sense of meaning can have positive effects on health. On the one hand, stressful situations were rated less negatively at the psychological level when a higher sense of meaning was experienced [14], [15]. On the other hand, a high sense of meaning led to better health behavior [16]. On a physical level, better treatment outcomes were achieved in the therapy of various diseases if a high sense of meaning was promoted in patients [15]. Despite these correlations, study results indicate that patients feel left alone with their questions of meaning within medical interactions [17], [18], [19].

In addition to findings from patient care, authors have described a sense of purpose as a preventive approach to protecting doctors from burnout and depression [20], [21]. Studies documented that a strong sense of purpose correlated with increased motivation and higher work engagement [4], [22], [23], [24]. It was also associated with a higher level of well-being, less time off work, generally improved job satisfaction, a better working atmosphere and higher efficiency [25], [26]. The following sources of meaning were identified for a positive sense of purpose in relation to the profession: the alignment of personal values with corporate values, the opportunity to take on responsibility and make decisions autonomously, active contribution to society, and personal development in a professional context [27], [28].

There is little research on the sense of purpose felt by medical students. Topics such as self-care, resilience and health have already been included in the international development of learning objectives [29], [30], [31], [32], [33], [34]. In Germany, a similar development has only taken place in recent years with the new National Competence-Based Learning Objectives for Medicine [https://nklm.de/zend/menu]. In this context, the topic of meaning is formulated as a health-relevant factor to be implemented as a goal [https://nklm.de/zend/menu], but it is questionable to what extent the topic is already addressed in existing medical curricula.

The aim of this study, the first of its kind to be conducted at two medical faculties, was to gain insights into the relation between questions of meaning for medical students in their own experience of meaning, in their studies and in dealing with questions of meaning from patients. The focus was on the intuitively described experience of meaning.

## 2. Methods

### 2.1. Study design and setting

An exploratory cross-sectional study was conducted with medical students from two different faculties at the beginning and end of the clinical study section. This approach was chosen to capture possible differences in the effect on students' sense of meaning that may arise from content orientation, study conditions, two different points in time in the study and the hidden curriculum [35] at the different locations. The medical faculty of Witten/Herdecke University (UW/H), a private institution, offers a model curriculum with early patient contact in the pre-clinical phase. The faculty is rather small with currently 84 students per semester. The faculty of the Technical University of Munich (TUM) offers a regular program with 175 students per semester. Following this online survey, which provides initial insights into the topic of meaning, in-depth interviews are scheduled to be conducted in a further study to gain a deeper understanding of this young field of research.

### 2.2. Instruments used

Since no questionnaire had previously been available to capture students’ questions about meaning, especially medical students, the research team developed the questions themselves in the winter semester of 2021/22. Based on the research interest, nine questions were developed in several meetings between FA (F. Albrecht), GL (G. Lutz) and CK (C. Kiessling) to explore the students’ perspectives. The questionnaire was then discussed and agreed upon with PM (P. Matcau), PB (P. Berberat) and NJ (N. Jedlicska) as part of an external audit. It contained six quantitative statements that were rated on a scale from 1=“disagree” to 5=“agree”. The instrument covered the following topics: the extent to which students had dealt with questions of meaning in their lives so far, whether they perceived their studies and clinical internships as meaningful, and whether their studies prepared them for their own questions of meaning or for confrontations they experienced in contact with patients. Three open-ended qualitative questions were used to gain initial insights into where students derive meaning from, how they deal with the confrontation with meaningful topics raised by patients or their relatives, and what they would like to see in their training. The collection of qualitative data enabled an open and flexible recording of students’ views [36]. In addition, sociodemographic data was collected on a voluntary basis, as can be seen in the questionnaire in attachment 1 . 

### 2.3. Data collection

The respective student deans’ offices contacted a total of 940 medical students (5^th^, 6^th^, 10^th^ and 11^th^ semesters; 700 at TUM, 240 at UW/H) by e-mail three times at two-week intervals with contentwise information about the study. Participation in the survey at the beginning of the 2022 summer semester was voluntary. An informed consent was obtained from participants. The data was collected anonymously using the online survey tool LimeSurvey.

### 2.4. Quantitative analysis 

RStudio 2022.02.2 Build 429 in conjunction with R Version 4.2.0 (The R Project for statistical computing, published 2022) was used for the statistical analysis. The data were analyzed descriptively. Group comparisons regarding location and study stage were performed using the Mann-Whitney U test. The significance level was set at p=0.05.

### 2.5. Qualitative analysis

Students’ free-text responses to the open questions were analyzed using a reflexive thematic analysis (RTA) based on Braun and Clarke with an iterative and deductive-inductive approach [37], [38]. As a first step, each answer was read by FA, GL and CK (phase 1) and then independently developed into initial inductive codes (phase 2). Independent coding served to increase the credibility and intersubjective comprehensibility [39], [40]. Coding units were units of meaning such as sentences or half-sentences. These first codings reduced the answers to the core statements in terms of content and created a first level of abstraction. Against the backdrop of the above questions an open analysis and topic development were carried out with a focus on the central importance of the various codes. In retrospect, the identified topics corresponded to the questions, but a new topic was also identified that was not covered by the questions. For these codes, superordinate topics were then discussed until agreement was reached (phase 3). In some cases, subtopics were identified in a subsequent run, for example when different characteristics of the experience were named in relation to sources of meaning. After the first topics and subtopics had been defined, an initial thematic mapping was carried out to check the internal homogeneity (assigned contents of a topic are similar) and external heterogeneity (topics differ from each other) in relation to the topics and subtopics. The topics, subtopics and thematic mapping were then discussed with GA (G. Atzeni), PM and PB in terms of fit and selectivity in the sense of an external audit (phase 4) and refined by adding a definition and further details to the topics (phase 5). The elaborations and preliminary definitions were discussed until a consensus was reached. Finally, the results of the evaluation were presented descriptively and visually (phase 6). In each phase, a reflection took place on personal, interpersonal, methodological and contextual factors that might influence the analysis of the data. For example, the team exchanged their own sources of meaning and also crises of meaning. Intergenerational aspects were also addressed, as the age range in the team was over 30 years. The different preferences for the organization of medical courses of study and affiliation with one of the two locations were also discussed and reflected upon, as were different disciplinary understandings of meaning. The Standards for Reporting Qualitative Research (SRQR) were used in the preparation of the manuscript [41].

### 2.6. Ethics vote

The study was reviewed by the UW/H Ethics Committee. No ethical or professional concerns were identified. The Ethics Committee of the TUM confirmed this vote.

## 3. Results

### 3.1. Description of the study population

A total of 111 participants completed the content-related questions in full (see table 1 (Tab. 1)). This corresponds to a response rate of 11.8%. Responses to the closed questions did not reveal any significant differences with regard to age and gender. In comparison to the total cohort of German medical students, the current surveys from 2021/22 show similar distributions. Of 105,275 students, 63.8% were female [42], while 64.4% of our cohort were female. The median average age of graduates in human medicine in 2021 was 26.1 [43].

### 3.2. Descriptive results of the quantitative data analysis 

92% (agree, rather agree) with only one exception stated that they had already addressed questions of meaning in their lives. Overall, 64% currently experienced their studies as meaningful. In terms of clinical block placements, 45% stated that these were meaningful. More than 30% were undecided in this regard. The question of whether the clinical placements involved a confrontation with questions of meaning from patients or their relatives was answered in the affirmative by around 59% of respondents. Only 18% disagreed with this statement. 47% of participants did not feel well prepared by their studies to deal with their own questions of meaning. In relation to the questions of meaning raised by patients, the figure was 56%. Around a third were undecided in this regard (mixed).

### 3.3. Group differences in terms of study stage

Students felt significantly less meaningful at the end of their studies compared to the beginning of their clinical training (see table 2 (Tab. 2)). At the same time, there was a significant decrease in the impression that the studies prepared them well for personal questions of meaning. 

### 3.4. Group differences in terms of location

When comparing the two study locations, UW/H students were more likely to report confrontations with patients’ questions about meaning than those at the TUM. Furthermore, UW/H students felt somewhat better prepared for personal and existential questions raised by patients and their relatives (see table 2 (Tab. 2)). 

### 3.5. Thematic analysis of free-text responses

**Figure 1:**
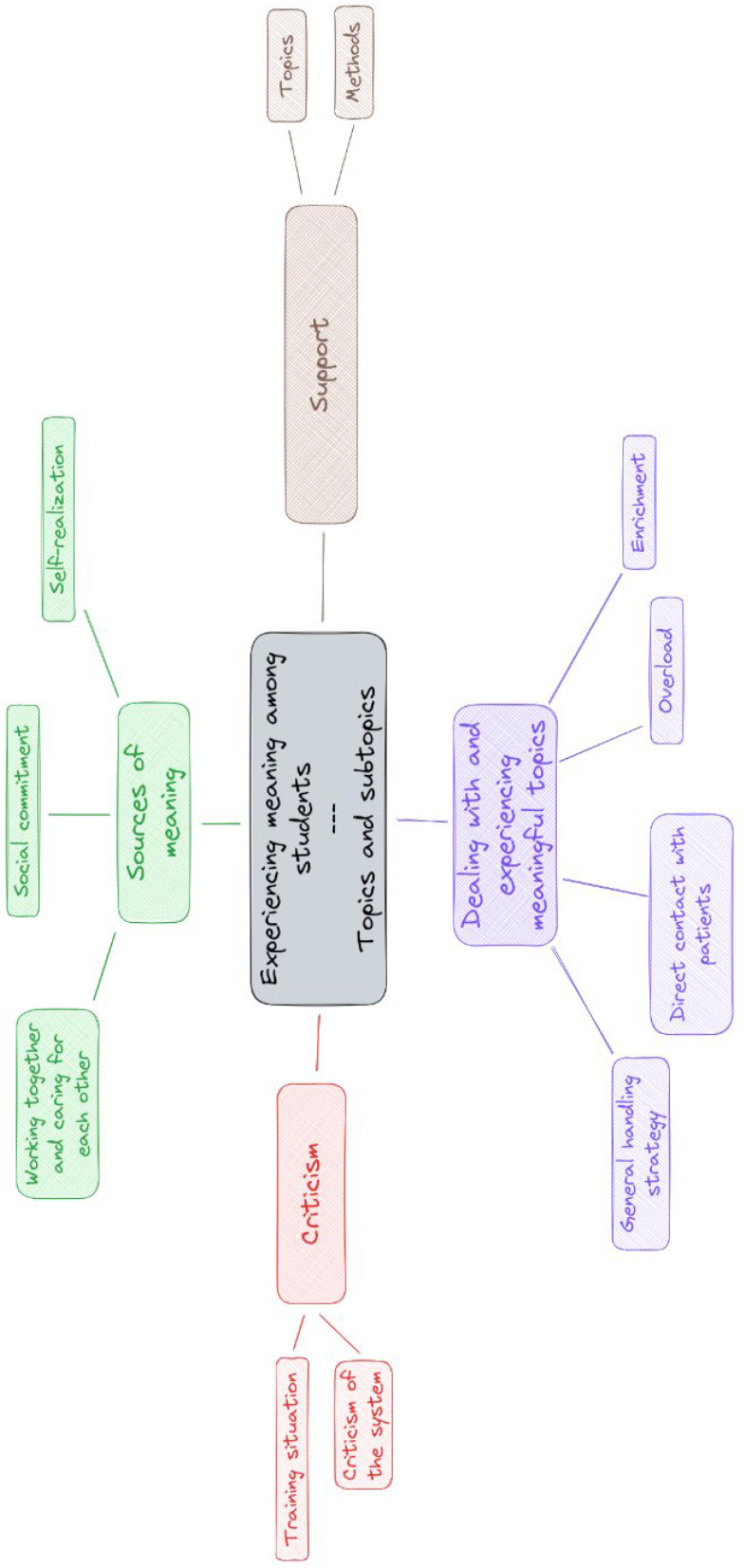
Insights into the meaning of medical students’ studies. Thematic analysis of the free text responses

Four topics with eleven precisely defined subtopics were extracted from the open comments. In the following, topics are marked as headings and subtopics with *single* quotation marks. Meaningful quotes can be found in table 3 (Tab. 3) and are partially included in italics in the results report. A complete list of all quoted comments can be found in attachment 2 . Each quoted or paraphrased free-text response was given a unique letter and number code. A thematic map is shown in figure 1 (Fig. 1).

#### Topic 1: Sources of meaning

The “common and caring togetherness” based *on a relationship of trust, respect and friendliness* [A1] was one of the most frequent sources of meaning, followed by “social engagement” in the context of medical care and interaction. Aspects of “self-realization” were also important, such as *personal growth* [C1], *pursuit of goals in life* [C2] and* the freedom to shape one’s life* [C3]. Aspects of self-transcendence were hardly mentioned. 

#### Topic 2: Dealing with and experiencing meaningful topics 

“General coping strategies” consisted of diverse and *constantly changing modes* [D1] of addressing topics of meaning. This included *openness towards others* [D2] and* regard for their perspectives* [D1], *joint discussions* [D3] and also theoretical approach to the topic via literature or university courses. 

In “direct interactions with patients”, some students preferred *to take on the challenge* [E1] and create an empathetic setting characterized by *active listening* [E2], *honesty* [E3] and the determination *to endure* [E4] meaningful topics. 

Some found these encounters and the opportunity *to get close to other people* [F1] “enriching” and were *grateful* [F1].

As an alternative to the strategy of daring to confront, respondents described the tactics of *repressing* [D4] both personal questions of meaning and those of patients, possibly because the confrontations were *“overwhelming”* [G1] and were perceived as frustrating. 

#### Topic 3: Need for support from the university

Various “requested topics” and “methodological” approaches were identified as support needs, most of which were limited to the curricular context. 

In terms of “content”, respondents wanted offers that *shed light on their own professional field* [H1] and the associated challenges, such as finding a good way to cope with increasing economization. In addition, needs were expressed to talk about* questions of meaning in medicine, stressful cases from everyday life* [H2] or ethical issues. 

“Methods” suggested by students to approach the issue were *interdisciplinary exchange* [I1], *experience reports* [I2] and* personality development* [I3], or ways to strengthen appreciative attitudes and empathy. Particular emphasis was placed on *practical experience* [I4], *adoption of different perspectives* [I5] and *supervision* [I6]. 

However, students felt that there was *hardly any time* for this [I7] due to an overloaded curriculum with a focus on clinical-theoretical subjects.

#### Topic 4: Criticism 

 The “current training situation” was frequently criticized: there should be *more practice overall* [J1] and *much less stand there and watch* internships [J2]. *The responsibility associated* [J3] with the profession was only insufficiently conveyed. Furthermore, it was criticized that teaching in its present form only insufficiently prepared students for questions of meaning and *dealing with their own values and goals* [J4] or for an adequate understanding of their role.

Further criticism was directed at the “health system”. *Economic and forensic aspects* [K1], *lack of time* [K2] and stress were mentioned together with doubts about the meaningfulness of the choice of profession and the option of *distancing oneself* [K3] from it.

## 4. Discussion

The aim of the study was to investigate the extent to which medical students in Germany are confronted with questions of meaning during their studies. About half of the students felt that their studies and practical placements were meaningful. The majority of students experienced questions of meaning in patient contacts during their internships, but did not feel sufficiently prepared for this. The majority of respondents had already dealt with questions of meaning. In this case, voluntary participation in a study on the topic of meaning in medical school can be assumed to be self-selection. Due to the lack of surveys, it is difficult to compare the high level of agreement with other student groups. Overall, the search for meaning in life seems to be a social trend that is reflected in a large number of popular scientific writings on “meaning”, “meaning crises” or “new work” [44], [45], [46], [47], [48]. Further reasons for the debate may be transition processes in coping with milestones and stress factors in adolescence [49], [50]. In addition, the considerable criticism in the free-text responses regarding education and the health system may provide a further explanation, as may the influences of the notoriously demanding curriculum [51], [52], as well as the potential effects of existential topics in patient contact. 

It was possible to identify sources of meaning for the students in the open comments. In particular, social aspects, one’s own actions for the benefit of others and aspects of self-realization were mentioned as sources of meaning. It is interesting to compare the sources of meaning that Tatjana Schnell and colleagues were able to identify in interview studies with a representative sample of the German population [53]. They found four overarching dimensions. Caring for each other and feeling connected was subsumed under the dimension of “we and well-being”. Sources of “self-realization” formed a second pillar. “self-transcendence” included aspects of placing one’s own existence and actions in a larger context. There was also the category of “order”, which was understood to mean tradition, morality, reason and down-to-earthness [4], [54]. While the most frequently mentioned source of meaning in our data, “we and well-being”, corresponds to a survey among the same age group, there was a striking difference in that aspects of order were hardly mentioned. On the one hand, a possible reason is that medical studies and the type of person who takes on a medical degree already have many order-creating characteristics and the search for it is no longer an issue. Another explanation may be the different approach of the survey. While Schnell and colleagues conducted interviews in which respondents had more time and space to reflect on their sources of meaning [4], our data source consisted of free-text responses, some of which were quite brief. 

There was a high level of agreement between students’ sources of meaning and the motives for studying medicine found in the literature. The greatest agreement was for caring motives, followed by aspects of self-realization and self-transcendence [55], [56]. Whether the above-mentioned peculiarities are specific to medical students remains open. Further work will be necessary to examine the specific sources of meaning and their proximity to motivations for specific studies.

A significant proportion of students felt that either medical studies in themselves or the practical approaches were not meaningful. While the experience of meaning in one’s work seems to be increasingly gaining social significance [4], [57], [58] and students, especially at the beginning of their studies, are characterized by a pronounced motivation and a high level of idealism [59], the experience of meaning appears to be already declining during training. Students at the end of their studies in particular showed lower levels of meaning-making, which suggests a decline during the clinical training phase. This is remarkable, as students’ meaningful aspects, such as the frequently described interactions with patients, increase during their studies. Research findings on the development of empathy, morality and patient-centeredness showed a comparable decline as clinical training proceeds. The causes of this decline in these constructs are discussed as including negative role models, educational experiences, the amount of learning material or the confrontation with barriers such as time pressure or the increasing market orientation of the system [59], [60], [61]. According to our data, comparable factors appear to have an effect on the sense of purpose. The incidence of criticism of the training situation and the system was striking, so that a close connection with the decreasing sense of purpose can be assumed. This may help to explain the critical development of why more and more young doctors no longer want to work full-time or are looking for ways out of the healthcare system [62], [63]. 

The majority of students have already been confronted with questions of meaning raised by patients or their relatives. This even affected students at the beginning of the clinical phase and is consistent with research findings that many patients want to address issues of meaning in medical treatment [17]. Furthermore, numerous studies emphasize the importance of experiencing meaning in relation to health outcomes in patients [14], [15], [16]. At the same time, patients’ existential questions often challenge the treating physicians. On the one hand, experience and targeted training are needed to perceive and tolerate the partly implicit topics [64], on the other hand, efforts to address them can trigger one’s own existential questions [65]. Although preparation for such conversations is actually anchored in the NKLM 2.0 as a learning objective for the first time [https://nklm.de/zend/menu], our data and the literature search conducted in advance of the study suggest that it has not yet been widely incorporated into the curricula. The frustrations or enrichments in the comments, depending on the course of the conversation, suggest that curricular amendments have the potential to promote student satisfaction. The differences in this respect between the two locations suggest that the better results are due to more longitudinal patient contact, which is already established in pre-clinical training at the UW/H. The increased experience apparently led to more successful coping strategies, such as a more conscious design of the discussion space and active listening as a method of changing perspectives and building relationships. Despite the differences, the preparation for questions of meaning at the UW/H was rated as only “mediocre”, so that these students might also benefit from curricular amendments. 

A large proportion of students expressed the feeling that they were inadequately prepared for questions of meaning. One possible explanation for this may be the lack of free time outside of their studies to deal with questions of meaning due to the increasingly extensive curricula. While students do not feel that university support is necessary for dealing with private questions of meaning, this seems to be different for career-related questions of meaning, as suggested by the wide range of proposals for curricular support. A one-size-fits-all approach does not seem to exist. Didactically different approaches to topics of meaning in different phases of education can be the right way here. The fact that UW/H students feel better prepared for questions of meaning may be due to the smaller size and the elective nature of the program. This offers space for various topics of interest to society as a whole and for personal development within the course of study, and seems to give students the freedom they need. With longitudinal thematic focuses such as “personal and professional development”, “clinical communication”, “interprofessional education” and “outpatient health care”, the UW/H also places greater emphasis on personal growth from the first semester onwards and offers more opportunities for personal needs to be met. The suggestion to make the training more student-centered and results-oriented has been under discussion for some time [66]. A deeper insight into the support needed and the exploration of possible influencing factors, such as different stages of development and different life paths of students, may create perspectives for optimized preparation. 

### Limitations

The study has a number of limitations. The sample is relatively small, with a response rate of 12%, so that generalization to other cohorts is limited, despite the consideration of two locations. Although the response rate is comparable to online surveys of students [67], a higher response rate would have been desirable. Another limiting factor is the voluntary participation in the survey. It cannot be ruled out that a disproportionate number of students who consider their sense of purpose to be meaningful took part in the survey. The collection of cross-sectional data only allows limited conclusions to be drawn about the course of events in the clinical phase. The qualitative analysis of the free-text responses can only be seen as a first step towards a deeper understanding of the sense of purpose in medical students. At the same time, the sample size offers a certain breadth of opinions and insights that can provide extremely helpful suggestions for conducting in-depth interviews [36]. Since our survey is the first survey on questions of meaning among medical students, further studies at other locations, including students of health care professions and within the framework of comprehensive longitudinal studies, would be desirable in order to validate and deepen our results.

## 5. Conclusion

Both the literature and the students’ reports emphasize the importance of experiencing meaning in the medical context. Initial indications received from students and their experiences underline the potential of university offerings to support students’ experience of meaning and to promote the long-term accompaniment of patients with questions of meaning. Study and framework conditions appear to be associated with a decline in experiencing meaning. With the current state of research, it is not possible to fully explain this observation. However, it would be important to identify evidence-based reasons for this in order to develop targeted interventions to support students.

The present survey provides an insight into the research of meaning in the context of medical studies which is only just beginning, and at the same time raises many questions: In what context and in what way do issues of meaning influence students? What are the effects of studies, practical placements and the experience of confronting patients’ issues of meaning on the experience of meaning? How can reflection and action processes be better and more individually supported in the context of guidance? In order to find answers to these questions, students will be interviewed in depth in a subsequent qualitative study. 

## Acknowledgements

We would like to thank Prof. Dr. T. Schnell, Professor at the MF Specialised University in Oslo, Norway, for her helpful support and feedback regarding research on the experience of meaning and her established meaning model. Our thanks also go to Christina Wagner for her very helpful support in translating the English version of the article. We would like to express our sincere thanks to all students who took part in the study. We would also like to thank the study deans who sent out the e-mails.

## Authors’ ORCIDs

- Felix Albrecht: [0000-0001-9927-7090]
- Gabriele Lutz: [0000-0001-5044-8485]
- Gina Atzeni: [0009-0002-9227-3980]
- Pascal O. Berberat: [0000-0001-5022-5265]
- Paula Matcau: [0009-0007-4119-6328]
- Nana Jedlicska: [0000-0001-8229-7845]
- Claudia Kiessling: [0000-0003-4104-4854]

## Competing interests

The authors declare that they have no competing interests. 

## Supplementary Material

Meaning questionnaire 2023 TUM UW/H

Thematic content of the free text responses with illustrative quotes organized by topic and sub-topic

## Figures and Tables

**Table 1:**
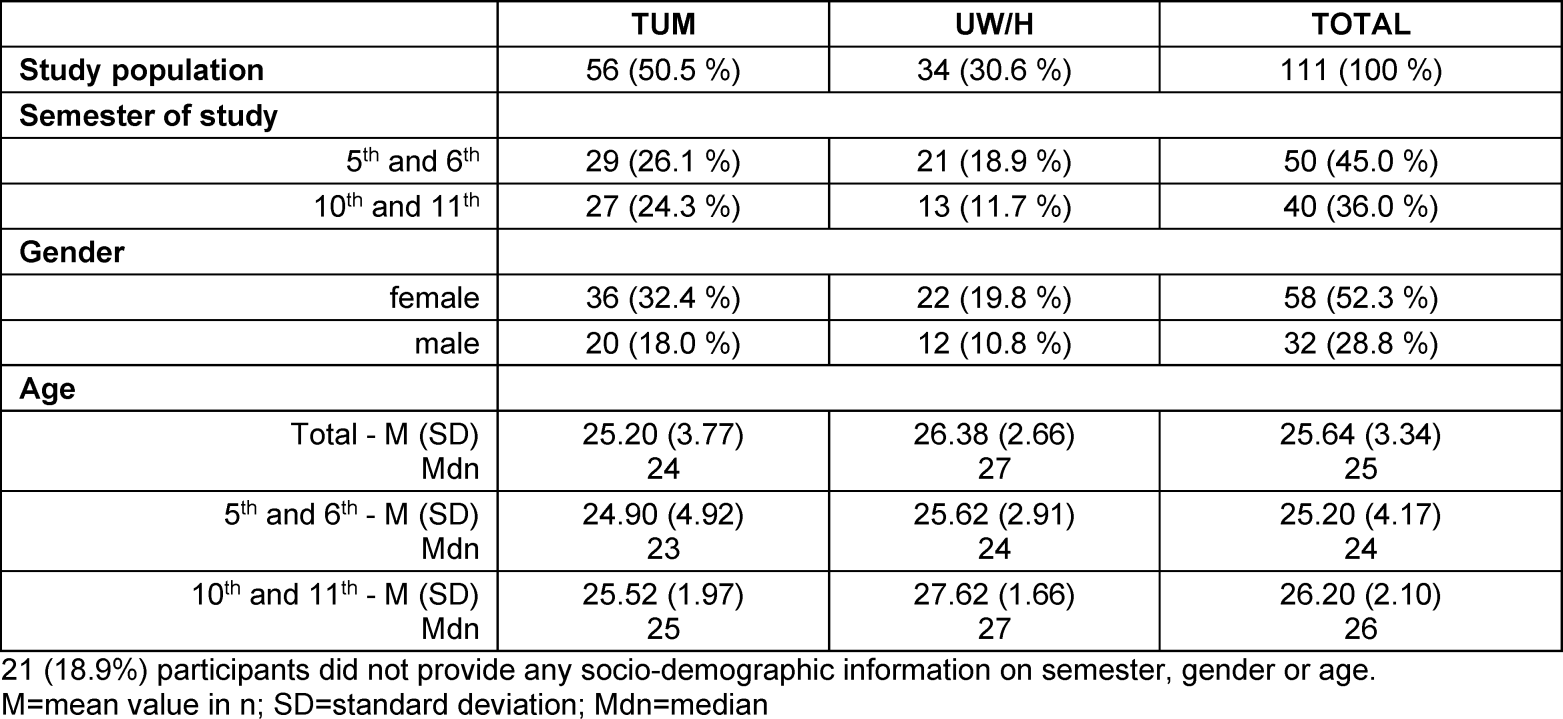
Detailed composition of the study population based on socio-demographic data (data in n and % in brackets)

**Table 2:**
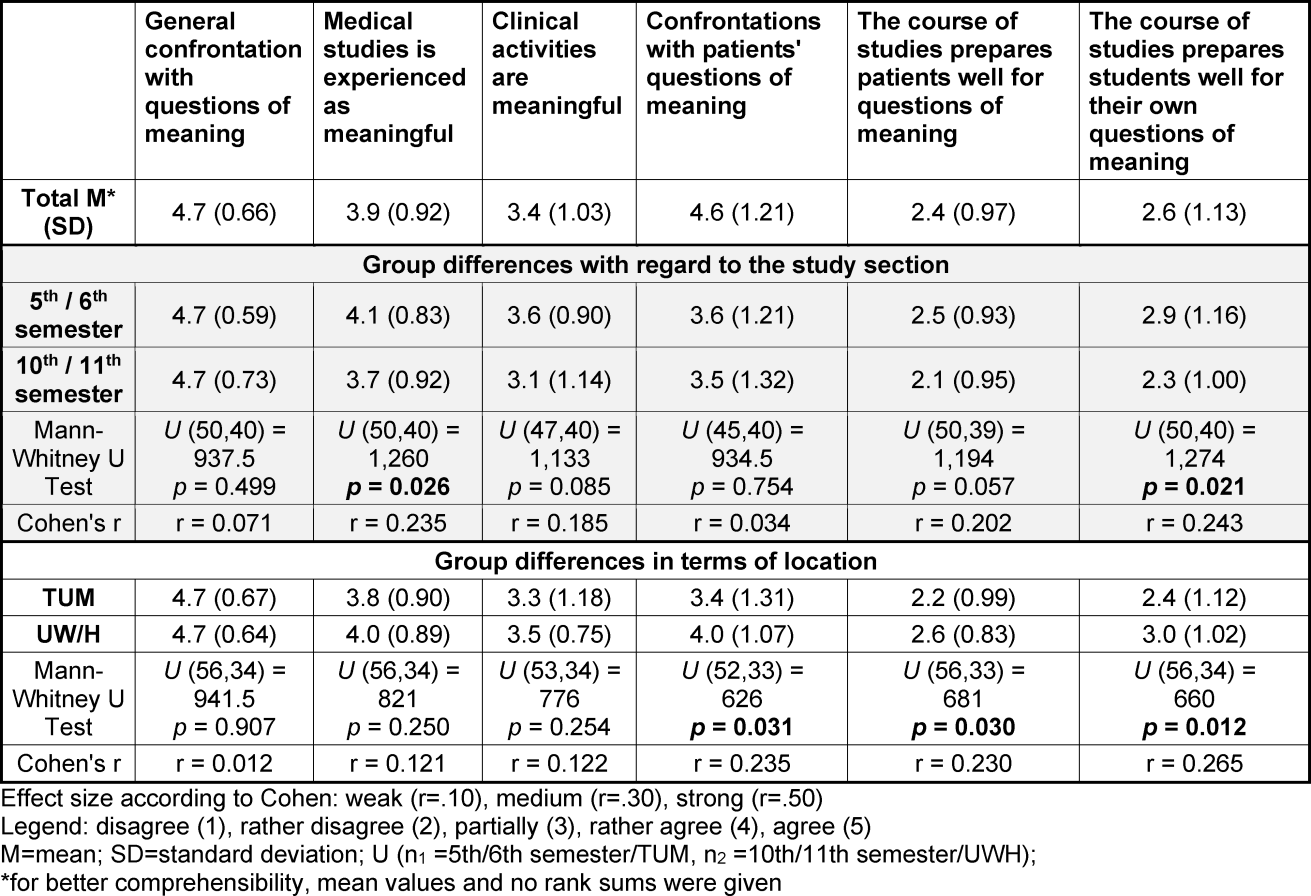
Mean values and differences according to study period and location comparison

**Table 3:**
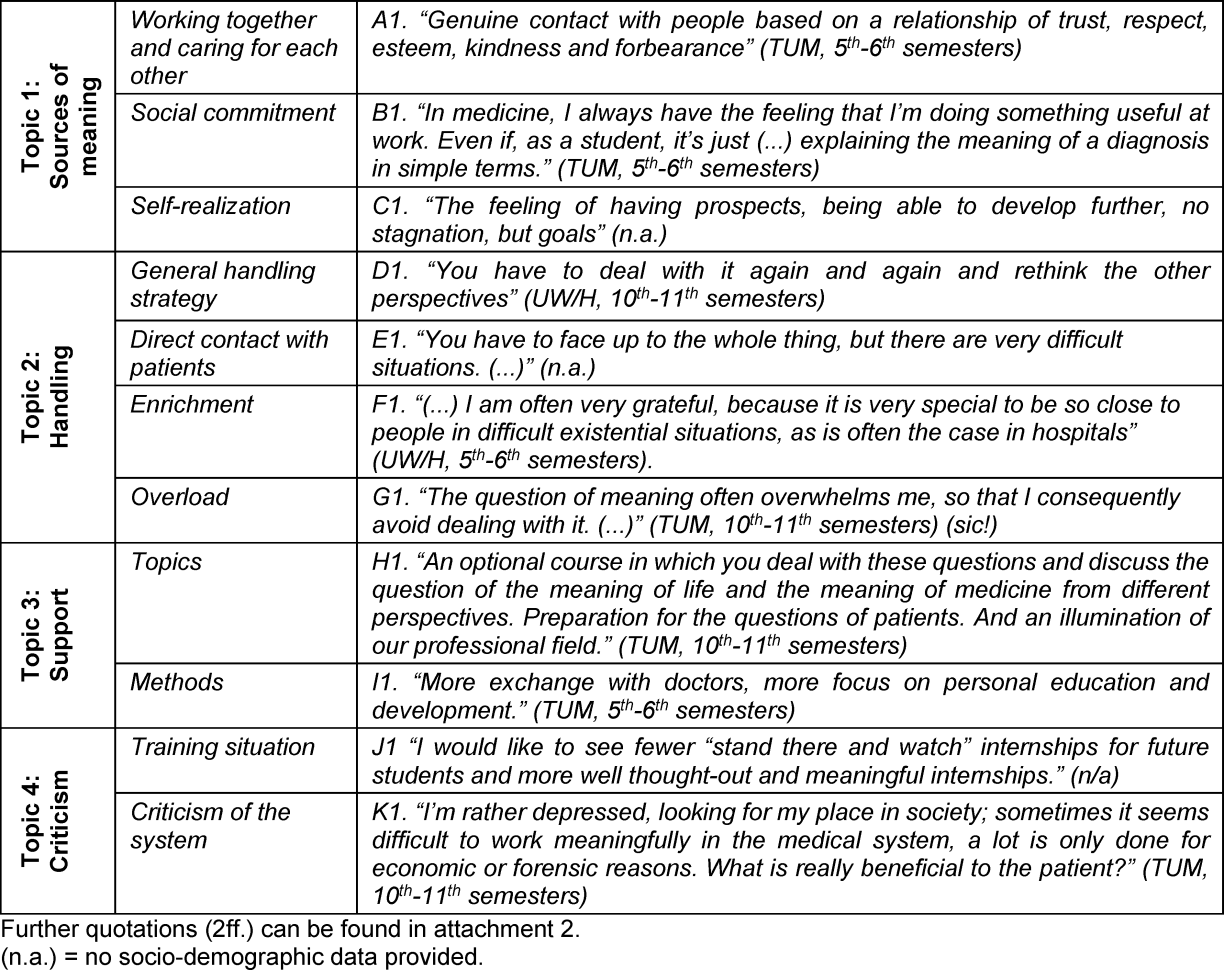
Exemplary selection of an illustrative quotation per subtopic
