# Supplementary material for: Insights into the meaning of medical students’ studies. An online survey at two medical faculties
Source: GMS J Med Educ. 2024 Sep 16;41(4):Doc45. doi: 10.3205/zma001700 (PMC11474645; doi:10.3205/zma001700)
Supplement: Meaning questionnaire 2023 TUM UW/H [file JME-41-45-s-001.pdf]

Attachment 1: Meaning questionnaire 2023 TUM UW/H

	agree	Rather agree	Partially	Rather disagree	disagree	No answer
I have already regularly dealt with my purpose in life.	☐	☐	☐	☐	☐	☐
I currently find my medical studies meaningful.	☐	☐	☐	☐	☐	☐
I find my previous medical work in my clinical blocks and internships particularly meaningful.	☐	☐	☐	☐	☐	☐
I regularly experience patients or their relatives asking me about the meaning of life.	☐	☐	☐	☐	☐	☐
How do you deal with it?	➔ FREETEXT ANSWER					
I feel well prepared for the questions patients ask about meaning through my studies.	☐	☐	☐	☐	☐	☐
I feel well prepared for my personal questions regarding my existence as a doctor through my studies.	☐	☐	☐	☐	☐	☐
What do you want from your studies in this regard?	➔ FREETEXT ANSWER					
What gives you a sense of purpose?<br>Professionally? Personally? In general?	➔ FREETEXT ANSWER					

Optional entry of socio-demographic data:
Status of clinical training phase (5th - 6th semesters / 10th - 11th semesters), age, gender (f/m/d), are you interested in participating in the interview study (title): (y / n), contact details (telephone / email)

Attachment 1 to Albrecht F, Lutz G, Atzeni G, Berberat PO, Matcau P, Jedlicska N, Kiessling C. *Insights into the meaning of medical students' studies. An online survey at two medical faculties.* GMS J Med Educ. 2024;41(4):Doc45. DOI: 10.3205/zma001700
