# Supplementary material for: Insights into the meaning of medical students’ studies. An online survey at two medical faculties
Source: GMS J Med Educ. 2024 Sep 16;41(4):Doc45. doi: 10.3205/zma001700 (PMC11474645; doi:10.3205/zma001700)
Supplement: Thematic content of the free text responses with illustrative quotes organized by topic and sub-topic [file JME-41-45-s-002.pdf]

Attachment 2: Thematic content of the free text responses with illustrative quotes organized by topic and *sub-topic*

Topic 1: Sources of meaning	
Working together and caring for each other	*A1. “Genuine contact with people based on a relationship of trust, respect, esteem, kindness and forbearance” (TUM, 5th-6th semesters)*<br><br>*A2. “For me personally, exchanging ideas with the people around me is meaningful, about what excites, worries and concerns us.” (n.a.)*<br><br>*A3. “Relationships with other people are particularly meaningful for me. Both professionally and personally, I exchange views with other people, make new contacts where possible and connect various of my contacts with each other to make new collaborations possible.” (TUM, 5th-6th semesters)*
Social commitment	*B1. “In medicine, I always have the feeling that I’m doing something useful at work. Even if, as a student, it was just that I was able to take away a person’s fear (...) to explain the meaning of a diagnosis in simple terms.” (TUM, 5th-6th semesters)*<br><br>*B2 “My studies and my job; supporting people in difficult times; implementing plans that make other people happy; the feeling of being needed!” (UW/H, 5th-6th semesters)*<br><br>*B3 “Doing a job that makes someone else’s life or the world a little bit better. Before that, I studied business administration and worked in an office. I never understood why I should work overtime or cause myself stress. So that the company can make a little more profit? So that a customer buys a few more things that they don’t actually need and are just being pushed on them? In medicine, I always have the feeling that I’m doing something useful at work. Even if, as a student, I was only able to ease someone’s anxiety because I could take a few more minutes than the doctor to explain the meaning of a diagnosis in simple terms.” (TUM, 5th-6th semesters)*
Self-realization	*C1. “The feeling of having prospects, being able to develop further, no stagnation, but goals” (n.a.)*<br><br>*C2. “Pursuing goals in life, no matter what area of life.” (UW/H, 5th-6th semester)*<br><br>*C3. “Not to have the feeling of spending your life doing something that you only feel others expect you to do = to be free in the way you organize your life.” (UW/H, 5th-6th semesters)*

Attachment 2 to Albrecht F, Lutz G, Atzeni G, Berberat PO, Matcau P, Jedlicska N, Kiessling C. *Insights into the meaning of medical students' studies. An online survey at two medical faculties.* GMS J Med Educ. 2024;41(4):Doc45. DOI: 10.3205/zma001700

Topic 2: Dealing with and experiencing meaningful topics	
General handling strategy	*D1. "You have to deal with it again and again and rethink the other perspectives" (UW/H, 10th-11th semesters)*<br><br>*D2. "(...) I accept that everyone understands their personal meaning differently and try to approach people as openly as possible in line with this." (TUM, 5th-6th semesters)*<br><br>*D3. "When it comes to my own questions of meaning, I usually deal with them alone in the evening first, and then usually open these questions up for discussion with my girlfriend or my circle of friends." (TUM, 5th-6th semesters)*<br><br>*D4. "Most of the time I suppress deeper considerations." (TUM, 10th-11th semesters)*
Direct contact with patients	*E1. "You have to face up to the whole thing, but there are very difficult situations. (...)" (n.a.)*<br><br>*E2. "I try to listen carefully to you, to understand the individual experiences and impressions that have led to the respective questions of meaning or crises of meaning. (...)" (n.a.)*<br><br>*E3. "I try to be honest and take the patients seriously. (...)" (UW/H, 10th-11th semesters) (sic!)*<br><br>*E4 "Approach, be there, endure." (UW/H, 5th-6th semesters)*<br><br>*E5. "But sometimes I also notice that I become numb or back away from these questions, because they are usually similar questions and emotions and situations." (n.a.) (sic!)*
Enrichment	*F1. "(...) I am often very grateful, because it is very special to be so close to people in difficult existential situations, as is often the case in hospitals." (UW/H, 5th-6th semesters)*<br><br>*Q2 "I find it meaningful (in the medical field) to have sustainably improved the lives of my patients. Not only to have improved the current suffering, but to have enriched each other through the exchange." (TUM, 10th-11th semesters)*
Overload	*G1. "Sometimes I get frustrated, but I still find the work very meaningful and important." (UW/H, 10th-11th semesters)*<br><br>*G2. "When I am confronted with questions of meaning in patient contact, I try to bring a little positive perspective to the whole thing in a rather clichéd way." (UW/H, 5th-6th semesters)*<br><br>*G3. "The question of meaning often overwhelms me, so that I consequently avoid dealing with it. (...)" (TUM, 10th-11th semesters) (sic!)*

Attachment 2 to Albrecht F, Lutz G, Atzeni G, Berberat PO, Matcau P, Jedlicska N, Kiessling C. *Insights into the meaning of medical students' studies. An online survey at two medical faculties.* GMS J Med Educ. 2024;41(4):Doc45. DOI: 10.3205/zma001700

Topic 3: Need for support from the university	
Topics	*H1. "An optional course in which you deal with these questions and discuss the question of the meaning of life and the meaning of medicine from different perspectives. Preparation for the questions of patients. And an illumination of our professional field." (TUM, 10th-11th semesters)*<br><br>*H2. "(...) and perhaps a room or contact person where stressful cases from everyday clinical practice can be discussed" (TUM, 10th-11th semesters)*<br><br>*H3. "I think the question of meaning comes up due to various working conditions that make it difficult not to be under constant stress. I think it would be good if there was more reality during our studies, that we work towards a stressful profession and that we later spend most of our time sitting behind a PC and not standing at a patient's bedside and can easily help them to heal." (UW/H, 5th-6th semesters)*
Methods	*I1. "More exchange with other disciplines so that medical students realize that they are not gods in white, but that others also make very important contributions." (n.a.)*<br><br>*I2 "More "stories from doctors", i.e. real experience reports (...)" (UW/H, 5th-6th semesters)*<br><br>*I3. "More exchange with doctors, more focus on personal education and development." (TUM, 5th-6th semesters)*<br><br>*I4. "Many patient contacts and discussions. Practical relevance. Learning from practical experience." (UW/H, 5th-6th semesters) (sic!)*<br><br>*I5. "More focus on training in perspective-taking (regarding patients, colleagues, other disciplines)" (n.a.)*<br><br>*I6 "Closer monitoring of patient contacts, e.g. through supervision." (TUM, 10th-11th semesters)*<br><br>*I7. "Sometimes there's not enough time because you're busy "keeping up" and paying attention." (TUM, 5th-6th semesters)*<br><br>*I8. "Discussion rounds; perspectives, role models." (TUM, 10th-11th semesters)*<br><br>*I9. "Many more medical interviewing courses and bedside training or a higher frequency of these." (TUM, 10th-11th semesters)*<br><br>*I10. "I am aware that it is not easy to develop a "fixed curriculum" for questions of meaning. But I would have liked to see these questions addressed more and perhaps to discuss them in small groups." (TUM, 5th-6th semesters)*

Topic 4: Criticism	
Training situation	*J1 "Simply more practice. We are in the clinic far too little. Why doesn't every student have to work in the clinic one or two days a week?" (n/a)*<br><br>*J2 "I would like to see fewer "stand there and watch" internships for future students and more well thought-out and meaningful internships." (n/a)*<br><br>*J3 "I sometimes get frustrated (...) when the implications that medical decisions can have for patients – and the responsibility that goes with them – are not communicated at university." (n.a.) (sic!)*<br><br>*J4. "I would like to see more space and guidance for personal development, for an active examination of one's own values and goals. I miss this component of being able to reflect on oneself and look beyond one's own ego and really perceive a situation or a person as they are with most of the doctors I deal with." (TUM, 10th-11th semesters)*
Criticism of the system	*K1. "I'm rather depressed, looking for my place in society; sometimes it seems difficult to work meaningfully in the medical system, a lot is only done for economic or forensic reasons. What is really beneficial to the patient?" (TUM, 10th-11th semesters)*<br><br>*K2. "More discourse on topics such as possible overuse in hospitals, what responsibility do we have, are there opportunities to take more time for patients, ..." (UW/H, 5th-6th semesters)*<br><br>*K3. "I have distanced myself more from my studies and withdrawn more from my fellow students and the university. (...) I have decided to only consider becoming a doctor if something fundamentally changes in medicine." (n.a.)*<br><br>*K4. "Hospital is not the place for questions of meaning. No room for discussion. No climate for intellectual debate. So I deal with it at home." (UW/H, 10th-11th semesters)*

Attachment 2 to Albrecht F, Lutz G, Atzeni G, Berberat PO, Matcau P, Jedlicska N, Kiessling C. *Insights into the meaning of medical students' studies. An online survey at two medical faculties.* GMS J Med Educ. 2024;41(4):Doc45. DOI: 10.3205/zma001700
